# Development and validation of a nomogram model for predicting residue of partially cystic thyroid nodules after ultrasound-guided ethanol and thermal ablation

**DOI:** 10.3389/fendo.2023.1128248

**Published:** 2023-02-28

**Authors:** Di Li, Xiaoer Zhang, Yutong Zhang, Tongyi Huang, Rui Zhang, Wenwen Zhou, Xiaoyan Xie, Ming Xu

**Affiliations:** Department of Medical Ultrasonics, Institute of Diagnostic and Interventional Ultrasound, The First Affiliated Hospital of Sun Yat-sen University, Guangzhou, China

**Keywords:** thermal ablation, ethanol ablation, partially cystic thyroid nodules, ultrasonography, nomogram

## Abstract

**Objectives:**

To develop and validate a nomogram model for predicting residue of partially cystic thyroid nodules (PCTNs) after ethanol and thermal ablation.

**Materials and Methods:**

From July 2015 to August 2022, a total of 97 patients (age 40.78 ± 12.61 years) with 107 treated benign PCTNs receiving ethanol and thermal ablation were enrolled. Pre-ablative laboratory test results and the ultrasound (US) and contrast-enhanced ultrasound (CEUS) features of lesions were collected. They were categorized into non-residue group and residue group according to the CEUS examination assessment after ablation. Univariate and multivariate logistic regression analysis were adopted to build a nomogram. The nomogram was validated by internal stratified fivefold cross-validation. The calibration, discrimination and clinical utility of the nomogram were investigated to assess the performance of the model.

**Results:**

Residue was reported in 30 out of 107nodules (28.0%). Multivariate logistic regression analysis revealed initial volume (OR=1.12, 95%CI 1.06-1.19) and presence of septum (OR=3.19, 95%CI 1.09-9.36) were predictors of residue of PCTNs. The nomogram developed by the above factors showed good calibration and discrimination. The area under the curve (AUC), sensitivity and specificity of this model were 0.832, 86.7% and 68.8%, respectively. When applied to internal validation, the model revealed good generalizability with stratified fivefold cross-validation in the cohort (mean AUC = 0.821).

**Conclusions:**

The nomogram model has good performance for predicting the residue of PCTNs undergoing ethanol and thermal ablation. This could play a role in the decision of treatment and follow-up in clinical practice.

## Introduction

Thyroid nodules are common in population, affecting up to 65% of them (1). Partially cystic thyroid nodules(PCTNs)are nodules with both solid and cystic components. With a malignancy rate less than 20%, most of PCTNs are benign and do not need intervention (2). But some of them can cause compressive symptoms or cosmetic problems and require treatment. Traditionally surgery was the standard solution of these problems but it still has some disadvantages, including scar formation, potential complications and damage of thyroid function (3). Thus, minimally invasive treatments are needed for benign PCTNs. Ultrasound(US)-guided thermal ablation, including radiofrequency ablation(RFA) and microwave ablation(MWA), was proved to be an effective substitute for surgery, which can decrease the nodule size significantly and simultaneously preserve the thyroid function and minimize complications (4–8). A meta-analysis showed that volume reduction rate (VRR) at 3, 6, and 12 months was 56.0%, 80.8% and 86.2% after RFA. And VRR after MWA was 53.9%, 74.9% and 80.0%, respectively. Significant decreased symptomatic and cosmetic scores were found after 6 and 12 months for both RFA and MWA (6). Besides, another study demonstrated that the incidence of hoarseness, hypothyroidism and postoperative pain were lower after thermal ablation compared to conventional thyroidectomy (9).

At the same time, for PCTNs, especially those with cystic components≥50%, simple aspiration of internal fluid with or without ethanol ablation (EA) was an initial recommended treatment (10). To enhance the efficacy, we adopted an EA-RFA combination therapy for these nodules in our study (11). The volume reduction rate (VRR) was commonly used as a measurement of the efficacy after ablation of thyroid nodules (12). However, just simple aspiration of the fluid and EA can achieve a great VRR but still reported a considerable rate of recurrence (13), which means VRR may not be an appropriate indicator of the prognosis of patients. The presence of solid component was considered a main cause of recurrence (14). For PCTNs that underwent RFA or MWA, the rate of recurrence was reported to be 5.6%-18.0% after ablation (15–17), which needs supplementary treatment. In the studies of Sim et al. (18) and Yan et al. (19), the incompletely treated residual vital tissue after ablation of thyroid nodules was independently related to recurrence. With the widespread use of contrast-enhanced ultrasound(CEUS), the residual vital tissue can be easily detected by CEUS after ablation and defined as enhanced area at ablative margin during both arterial phase and venous phase. Similar concept has been generally used to assess disease progression in image-guided tumor ablation (20). Therefore, the presence of residual vital tissue can be a preferable parameter of efficacy, compared to VRR. As we know, the pre-ablative prediction of the residual vital tissue is still unexplored for PCTNs, which is important for the decision-making before the management of PCTNs and follow-up strategy after ablation.

Therefore, the aim of this study was to construct a prognostic nomogram model based on pre-ablative parameters to predict the residual vital tissue after ablation of PCTNs.

## Materials and methods

The protocol of this retrospective study was approved by the Institutional Review Board of the First Affiliated Hospital of Sun Yat-Sen University. Since it was a retrospective study, informed consent of the patients could not be obtained, but all patients has signed informed consent before the ablation and CEUS.

### Patients

From July 2015 to August 2022, a total of 97 consecutive PCTNs patients (age 40.78 ± 12.61 years, with 107 treated nodules) were enrolled. The inclusion criteria were as follows: (1)patients with benign nodules confirmed cytologically by ultrasound-guided fine-needle aspiration (FNA); (2)nodules without suspicious echographic features of malignancy; (3)patients with partially cystic thyroid nodules(10%≤cystic components ≤ 90%); (4)patients who complained about compression or cosmetic problems, or with anxiety about malignant transformation; (5) patients who refused or ineligible for surgery; (6)patients with a post-ablative follow-up time≥1 month. The exclusion criteria were: (1) malignant nodules on US imaging; (2) cytologically confirmed malignancy; (3) patients with totally solid or cystic thyroid nodules; (4) patients allergic to ultrasonic contrast agent; (5)patients with severe cardiopulmonary disfunction or coagulation disorder; (6) patients with incomplete data.

### Pre-ablation assessment

**Figure 1:**
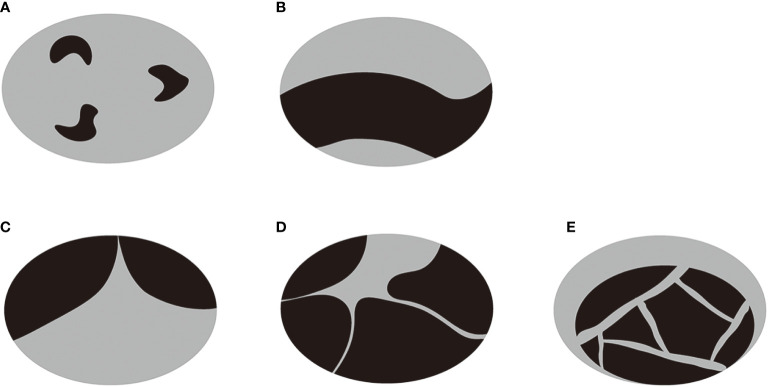
A diagram of the classification of PTCNs. **(A)** One or more small cysts were scattered among continuous solid component. **(B)** Discontinuous solid components were adhered to the wall separated by cystic component. **(C)** There was one septum in the largest cyst. **(D)** There were several septa in the largest cyst with a radial pattern. **(E)** There were several septa in the largest cyst with a random pattern. The type A and B were non-segregated nodules, and type C, D and E were segregated nodules.

Before ablation, patients underwent US and CEUS examination, FNA and laboratory examination. All CEUS examinations were carried out by multiple senior sonographers with more than 5 years of experience in thyroid examination. US and CEUS examination were performed using a Toshiba Aplio i900 Ultrasound System (Canon, Tokyo, Japan) or a Philips iU22 Ultrasound System (Philips Healthcare, Bothell, WA) with a linear multifrequency probe. Nodule volume, location, adjacent structures, proportion of cystic component, echogenicity of cystic component, vascularity, presence and type of septum and enhancement of solid component were assessed on US and CEUS. The nodule volume was calculated using three orthogonal diameters (the largest one(a) and two diameters perpendicular to it (b and c)): V = πabc/6. The location of each nodule was classified as right lobe, left lobe and Isthmus. The adjacent structures (<2mm) were classified as trachea, esophagus, large cervical vessels (carotid artery and jugular vein), recurrent laryngeal nerve and none of the above. The proportion of cystic component was measured using software ImageJ on the largest section of the nodule. The echogenicity of cystic component was classified as echoless and mixed echo. Nodule vascularity was evaluated for the solid component according to the Alder grading system of blood flow: grade 1 (absent): no blood flow visualized; grade 2 (minimal): blood flow of one or two pixels; grade 3 (moderate): a main vessel or several small vessels; grade 4 (marked): four or more vessels (21). Septum was defined as one or more bands (thickness < 2mm, length > 2mm) in the largest cyst with both fixed ends, reported as absent or present. According to the type of septum and distribution of cystic and solid components, the nodules were further classified into 5 types: type A: one or more small cysts scattered among continuous solid component; type B: discontinuous solid components adhered to the wall separated by cystic component; type C: one septum in the largest cyst; type D: several septa in the largest cyst with a radial pattern; type E: several septa in the largest cyst with a random pattern (**Figure 1**). The type A and B were non-segregated nodules, and type C, D and E were segregated nodules. The process of CEUS examination consists of arterial phase (0–30s after the administration of contrast agent) and venous phase (30–120s after contrast agent injection). Enhancement of solid component in the two phases was evaluated as hypo-, iso- and hyper-enhanced compared to surrounding normal tissue. Nodule location, adjacent structures, echogenicity of cystic component, vascularity, presence and type of septum and enhancement of solid component were assessed by 2 doctors with more than 5 years of experience in thyroid examination independently. If there was difference between them, a third doctor with more than 10 years of experience in thyroid examination would re-assessed the characteristics and made the final decision.

Laboratory results collection included the level of thyroid stimulating hormone (TSH), serum free triiodothyronine (fT3), serum free thyroxine (fT4), complete blood count and coagulation tests. Additionally, all patients underwent electrocardiogram, laryngoscopy and chest X-ray or CT before ablation.

### Ablation procedures

All invasive operations were performed by 2 interventional doctors with more than 5 years of experience in ablation. The operator adopted simple aspiration of fluid and ethanol ablation as a combined therapy of thermal ablation when (1): the proportion of cystic component was more than 50% and the type of septum was not type A; (2) the volume of cystic component was estimated to be more than 10ml on the largest section and its perpendicular section. For nodules with small cysts, only thermal ablation is enough to damage the structure of cyst wall. However, for nodules with large cysts, thermal ablation is not enough so simple aspiration and ethanol ablation are needed to damage the wall completely. Patients were requested to assume a supine position with the neck extended. Intravenous analgesia plus local anesthesia was used during ablation. The operator first aspirated the fluid in the capsule and then repeatedly flushed the capsule cavity with anhydrous alcohol to complete the ethanol ablation. The dosage of alcohol was approximately 50% of the aspirated fluid volume. Then if the distance between the target area and the adjacent important structures was less than 2mm, 5% glucose solution would be introduced to form hydrodissection and separate the thyroid gland and adjacent important structures. In RFA, a Cool-tip RFA system (Covidien, Mansfield, Mass) with a 17-gauge electrode and a 1-cm active tip would be used percutaneously in the procedures. With real-time US guidance, the ablation was performed in a trans-isthmic approach, using moving-shot technique. The ablation power was set as 40w. High temperature produced necrosis in target nodule. The instrument shut down when the impedance increased to a certain degree. In MWA, the procedures were similar. A KY-2450B MWA system (Kangyou Medical, Nanjing, China) with a 16-gauge needle antenna and a 1.1-cm active tip was inserted into the nodule in a trans-isthmic way with the guidance of US. The ablation power was 30w. As heat built up at the target area, microbubbles would appear on US images. The procedure was continued by moving the antenna towards the other parts of the nodule. The procedure reached the end when the nodule appeared completely hyperechoic. Then we drew back the electrode and cauterized the pathway to avoid bleeding and seeding.

The technical variables, including power applied, number of electrode and times of power release, and duration of ablation, were recorded during the procedure.

### Post-ablation evaluation

**Figure 2:**
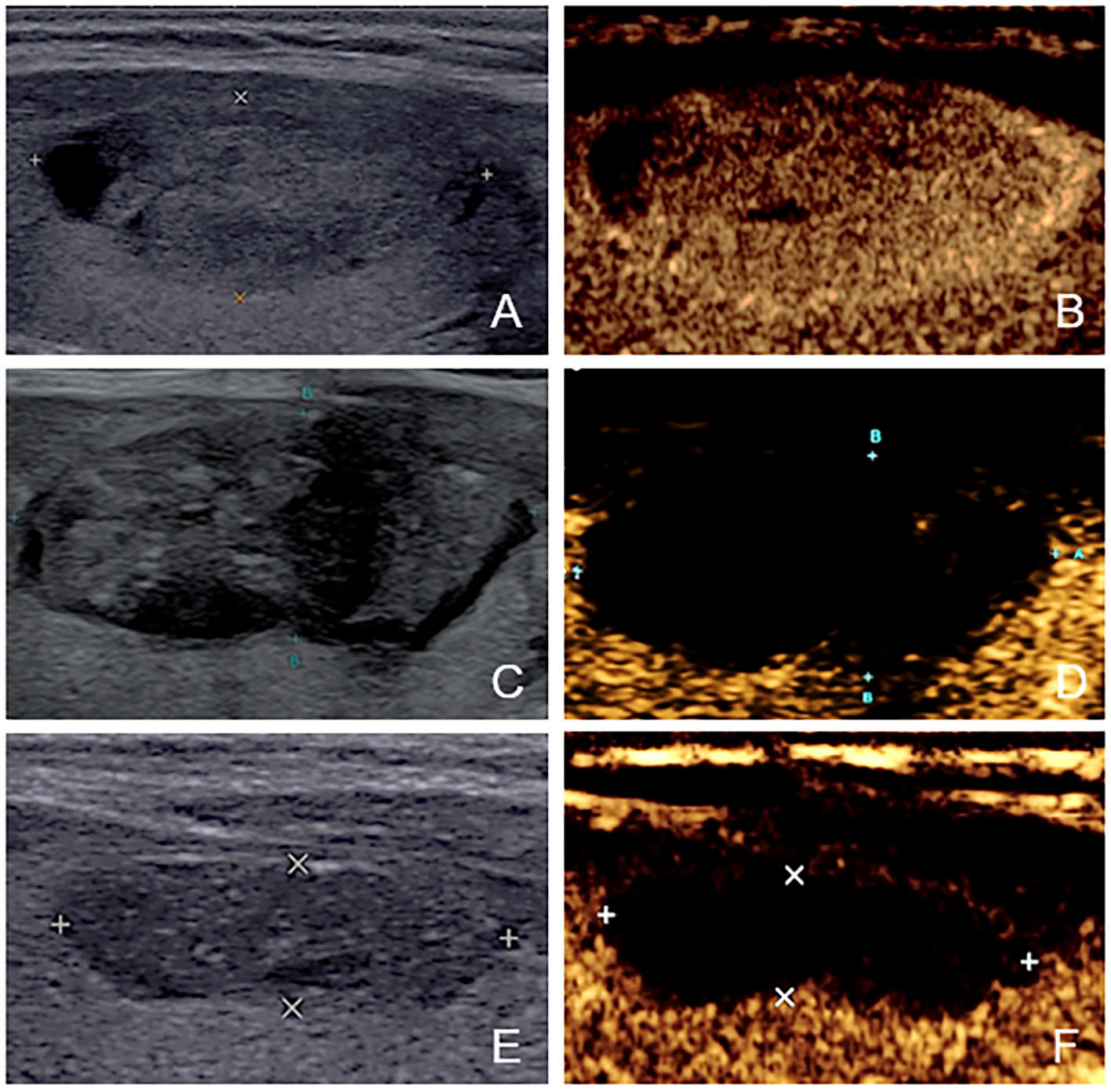
The US and CEUS feature of a case in non-residue group—a 41-year-old female. **(A, B)** Before ablation, the initial volume of the target nodule was 3.22 ml. There was no septum inside the nodule. Her residue rate according to the nomogram was 8.1%. **(C, D)** At one-month follow-up, no enhanced area was observed at the ablative margin under CEUS. **(E, F)** At four-month follow-up, the volume was 0.62ml, and VRR was 80.75%.

**Figure 3:**
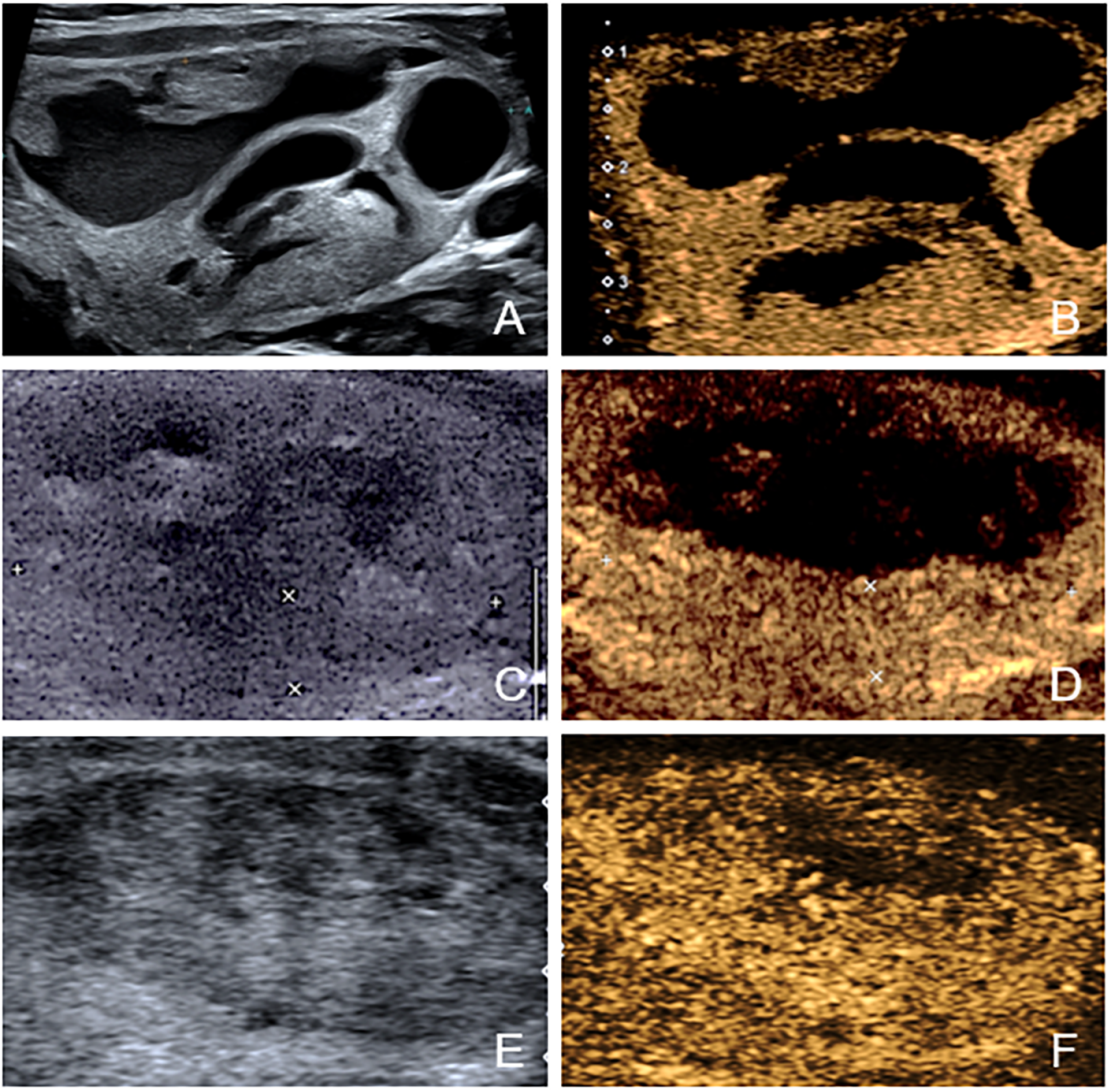
The US and CEUS feature of a case in residue group—a 34-year-old female. **(A, B)** Before ablation, the initial volume of the target nodule was 21.21 ml. There was obvious septum in the nodule. Her residue rate according to the nomogram was 58.5%. **(C, D)** At one-month follow-up, an enhanced area (about 3.0cm×0.6cm) was observed at the ablative margin under CEUS. **(E, F)** At four-month follow-up, the volume was 12.90ml, and VRR was 39.18%.

After ablation, CEUS was performed immediately and one day later to assess ablated field. Additionally, the level of thyroid stimulating hormone (TSH), serum free triiodothyronine (fT3) and serum free thyroxine (fT4) were tested within 24 hours after treatment. The same follow-up routine was performed 1 month or later after ablation. Any short- and long-term complications were recorded. On CEUS, the ablated nodule would be presented as non-enhanced area if the ablation was complete and there was no residual vital tissue. In contrast, if enhanced area at the ablative margin was observed at any follow-up≥1month later, residual vital tissue was considered to be present. To accurately judge the ablative margin, the sonographers should scan the nodules on three perpendicular planes dynamically and consider 3 aspects comprehensively. First, they compared the images of the nodules after ablation with those obtained before ablation. If the original nodule was completely non-enhanced, it would be considered to be non-residual. Otherwise, residue may exist. Second, the completely ablated area is usually a regular and smooth ellipse. In contrast, the residual part usually has an inward tendency and turns the area to an irregular part with projections on the margin. Third, they evaluated the nodules combining US and CEUS. The solid part of most nodules has mixed echo that can be distinguished from normal thyroid parenchyma. For the nodules that were isoechoic on US and iso-enhanced on CEUS, they usually have a clear boundary with the surrounding normal thyroid parenchyma. That’s to say, as long as the nodules can be detected before ablation, the residual part can also be distinguished from normal tissue. We did not include the follow-up within one month because the non-enhanced portion could not represent the actual necrotic tissue immediately (22). According to the results of follow-up CEUS examinations, the nodules were categorized into non-residue group and residue group. **Figures 2**, **3** shows the representative cases of the two groups.

### Statistical analysis

All statistical analyses were performed using SPSS version 26.0 software (IBM Corp. Released 2019. IBM SPSS Statistics for Windows, Version 26.0. Armonk, NY: IBM Corp.) and R software version 4.2.1. Continuous variables were presented as mean ± SD. Mann–Whitney U test was used to compare age, level of TSH, FT3, FT4, volume and proportion of cystic component between the two groups. Categorical variables were reported as frequencies (percentages) and Chi-square test was used to compare them between two groups. Then univariate and multivariate logistic regression analysis were constructed to confirm whether independent variables existed. Based on these independent factors, a nomogram was constructed to predict the probability of residue after ablation. The performance of the model was tested by calibration, discrimination and clinical utility. The Hosmer–Lemeshow test was used to assess the goodness of fit of this model. A calibration curve was also produced to visualize the calibration of the nomogram. The discrimination was presented as C-index, equivalent to the area under the receiver operating characteristic (AUROC) curve. The Youden index was used to identify the best cutoff value of the ROC curve. To evaluate the clinical usefulness of the model, decision curve analysis (DCA) was constructed with 1,000 bootstrap resamples of the study group. Additionally, internal stratified fivefold cross-validation was performed to demonstrate the generalizability. All the statistical analyses were two-tailed and a P value less than 0.05 was considered statistically significant.

## Results

Totally 97 patients (90 females, 17males, age 40.78 ± 12.61years) with 107 PCTNs were included in this study (**Table 1**). 89 out of these 97 patients had 1 nodule, 6 patients (6.2%) had 2 nodules and 2 patients (2.1%) had 3 nodules. Among all patients, 3 patients had hyperthyroidism while the other had normal thyroid function. The initial largest diameter and volume of the nodules were 3.38 ± 1.23 cm and 11.20 ± 11.11ml, respectively. The proportion of cystic component was 41 ± 27%. About half of the nodules had septum. Among the non-segregated nodules, 40 nodules were classified as type A and 10 nodules as type B. As for segregated nodules, 26, 14 and 17 nodules were respectively classified as type C, D and E. The mean follow-up was 16.0 months (range from 1 to 82 months) after ablation.

**Table 1:** The basic and ultrasound characteristics.

Variables	Data
Clinical parameters	
No. of patients/nodules	97/107
Age (years)	40.78 ± 12.61
Sex (F/M)	90/17(84.11/15.89)
Thyroid function	
Euthyroidism/Hyperthyroidism	94/3(96.91/3.09)
Ultrasonic parameters	
Location	
Right lobe/Left lobe/Isthmus	51/52/4(47.66/48.60/3.74)
Largest diameter (cm)	3.38 ± 1.23
Initial volume (ml)	11.20 ± 11.11
Proportion of cystic component	0.41 ± 0.27
Septum No/Yes	57/50(53.27/46.73)
Type A/B/C/D/E	40/10/26/14/17(37.38/9.35/24.30/13.08/15.89)
Vascularity	
Grade 1/2/3/4	13/44/27/23(12.15/41.12/25.23/21.50)
Echogenicity of cystic component Echoless/mixed echo	64/43(59.81/40.19)
Arterial phase enhancement hypo-/iso-/hyper-enhanced	5/91/11(4.67/85.05/10.28)
Venous phase enhancement hypo-/iso-/hyper-enhanced	30/70/7(28.04/65.42/6.54)

Data are presented as mean ± SD or number of nodules (percentages).

27 out of 97 patients (27.8%) reported adverse events after ablation. Mild local pain was the most common side effect that occurred in 17 patients (17.5%). Slight swelling of the neck can be seen in 13 patients (13.4%). 4 patients and 1 patient, respectively, experienced dysphonia and dysphagia. Most of these symptoms resolved within 3 days after treatment with oral analgesics, neurotrophic methycobal and dexamethasone. One patient suffered from dysphonia that resolved after 3 months.

Residue was found in 30 out of 107nodules (28.0%). Among them, 2 patients received a second ablation 3 and 5 months respectively after the initial ablation and reached complete ablation. The principle of supplementary ablation was based on patient’s desire and presence of compression or cosmetic problems. The comparison of the two groups was presented in **Table 2**. The initial volume (19.7 ± 12.9 ml *vs* 7.9 ± 8.3 ml, *P <*0.001) was significantly larger in the residue group than that in the non-residue group. 76.7% (23/30) of the nodules in the residue group had septum, while only 44.2% (34/77) in the non-residue group had septum (*P*=0.002). The ablation time and energy were also statistically related to residues. Longer ablation time and larger ablation energy were related with higher possibility of residue. This is because when the initial volume of the nodule was larger, the operators would decide to ablate for longer time with larger energy. Because we aimed to construct a pre-ablative model for better decision of treatment before ablation, we did not select ablation time and energy as independent factors in our model. For other parameters collected in this study, there were no significant difference between the two groups (all *P >*0.05).

**Table 2:** The comparison between the non-residual and residual groups.

Variables	Non-residual group	Residual group	*P* value
Pre-treated parameters			
Age (years)	41.2 ± 12.4	39.6 ± 13.3	0.492
Sex (Male/Female)	12 (15.6%)/65 (84.4%)	5 (16.7%)/25 (83.3%)	1.000
TSH (uIU/mL)	1.6 ± 1.2	1.3± 1.0	0.101
FT3 (pmol/L)	4.8 ± 0.6	5.1 ± 1.4	0.317
FT4 (pmol/L)	11.4 ± 2.0	12.1 ± 4.7	0.854
Initial volume(ml)	7.9 ± 8.3	19.7 ± 12.9	<0.001
Adjacent critical structures (No/Yes)	5 (6.5%)/72 (93.5%)	1 (3.3%)/29 (96.7%)	0.865
Septum (No/Yes)	43 (55.8%)/34 (44.2%)	7 (23.3%)/23 (76.7%)	0.002
Non-segregated type(type A/B)	36 (83.7%)/7(16.3%)	4 (57.1%)/3(42.9%)	0.262
Segregated type (type C/D/E)	19 (55.9%)/7(20.6%)/8(23.5%)	7 (30.4%)/7(30.4%)/9(39.1%)	0.165
Proportion of cystic component	0.4 ± 0.3	0.4 ± 0.2	0.981
Echogenicity of cystic component (Echoless/mixed echo)	50 (64.9%)/27 (35.1%)	14 (46.7%)/16 (53.3%)	0.083
Vascularity(Grade 1/2/3/4)	12 (15.6%)/32 (41.6%)/ 16 (20.8%)/17 (22.1%)	1 (3.3%)/12 (40.0%)/ 11(36.7%)/6 (20.0%)	0.181
Arterial phase enhancement(hypo-/iso-/hyper-enhanced)	4 (5.2%)/67 (87%)/ 6 (7.8%)	1 (3.3%)/24 (80.0%)/ 5 (16.7%)	0.458
Venous phase enhancement(hypo-/iso-/hyper-enhanced)	24 (31.2%)/49 (63.6%)/ 4 (5.2%)	6 (20.0%)/21 (70.0%)/ 3 (10.0%)	0.395
Ablation-related parameters			
Hydrodissection (No/Yes)	41 (53.2%)/36 (46.8%)	15 (50.0%)/15 (50.0%)	0.763
Ablation type(RFA/MWA)	71 (92.2%)/6 (7.8%)	25 (83.3%)/5 (16.7%)	0.316
Ethanol ablation(No/Yes)	56 (72.7%)/21 (27.3%)	21 (70.0%)/9 (30.0%)	0.778
Ablation time(min)	13.2 ± 8.5	23.5 ± 10.2	<0.001
Ablation energy(kJ)	31.2 ± 20.6	54.5 ± 24.8	<0.001

Continuous variables were presented as mean ± SD and categorical variables were reported as frequencies (percentages).

**Figure 4:**
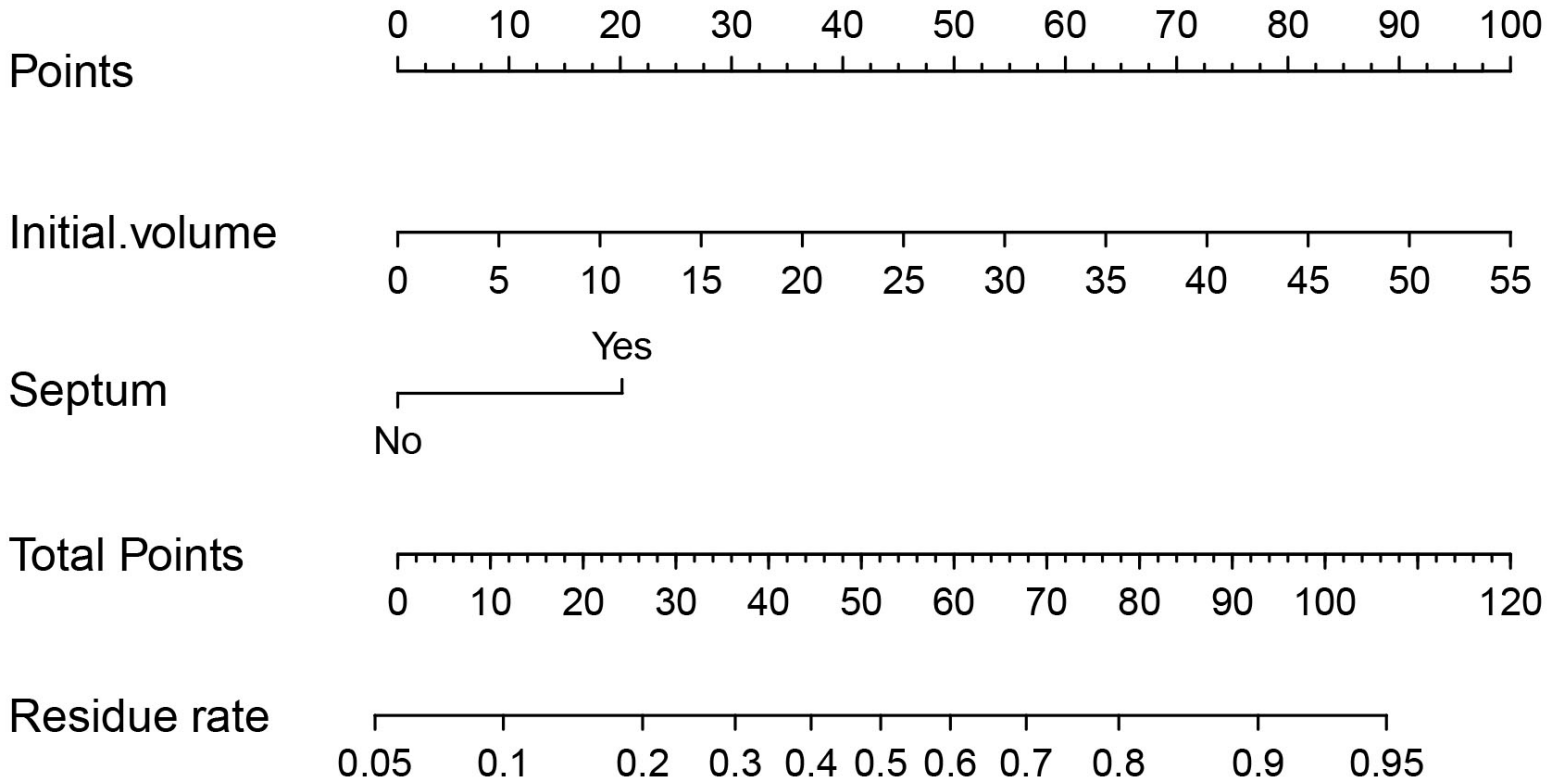
A nomogram to predict residue rate of PCTNs after ablation.

**Figure 5:**
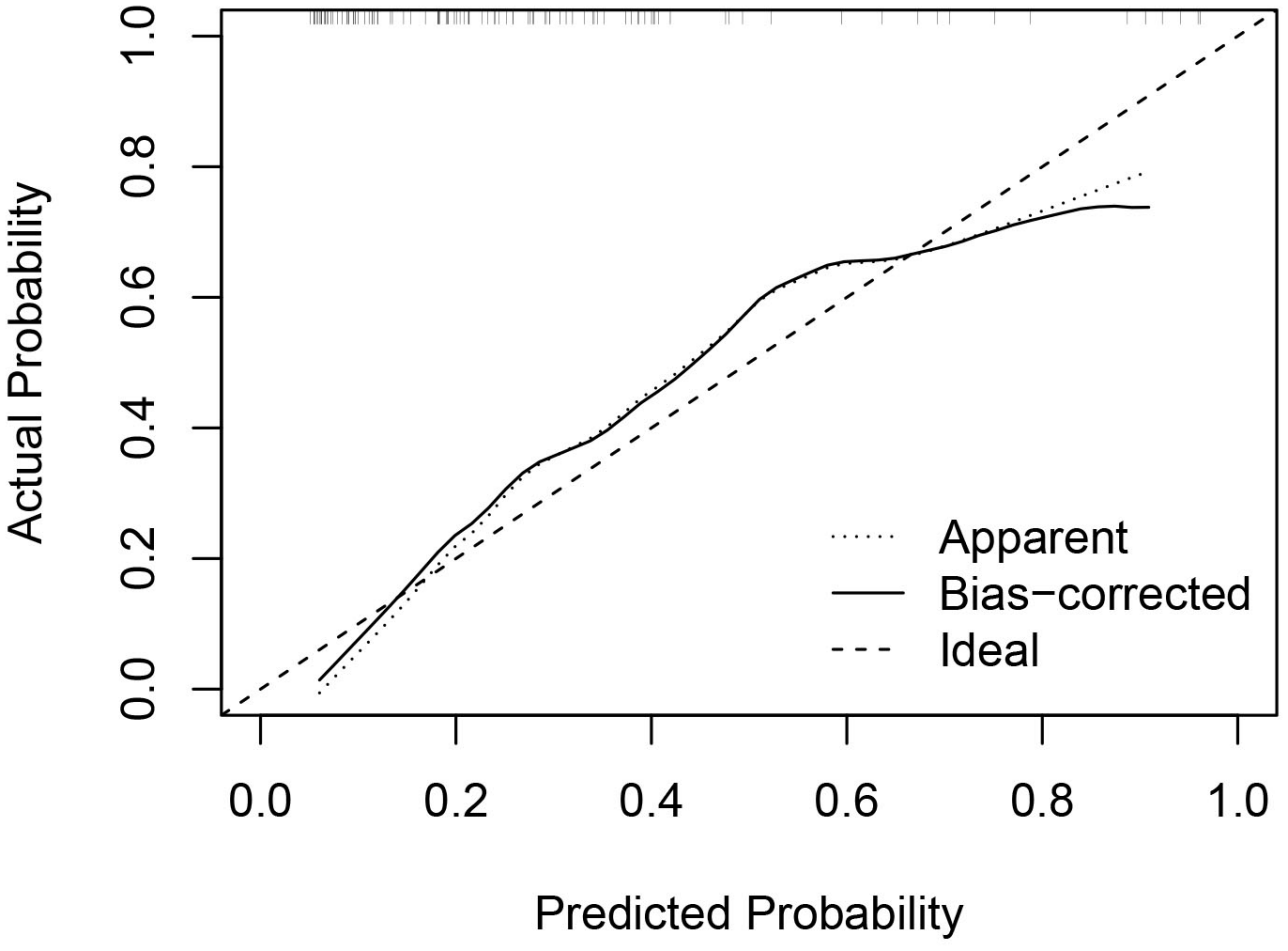
Calibration curve of the nomogram. The x axis represented the predicted probability of residue and the y axis represented the actual probability. The diagonal dashed line means an ideal model that can perfectly predict the residue. The solid line indicated the apparent predict performance of the nomogram.

**Figure 6:**
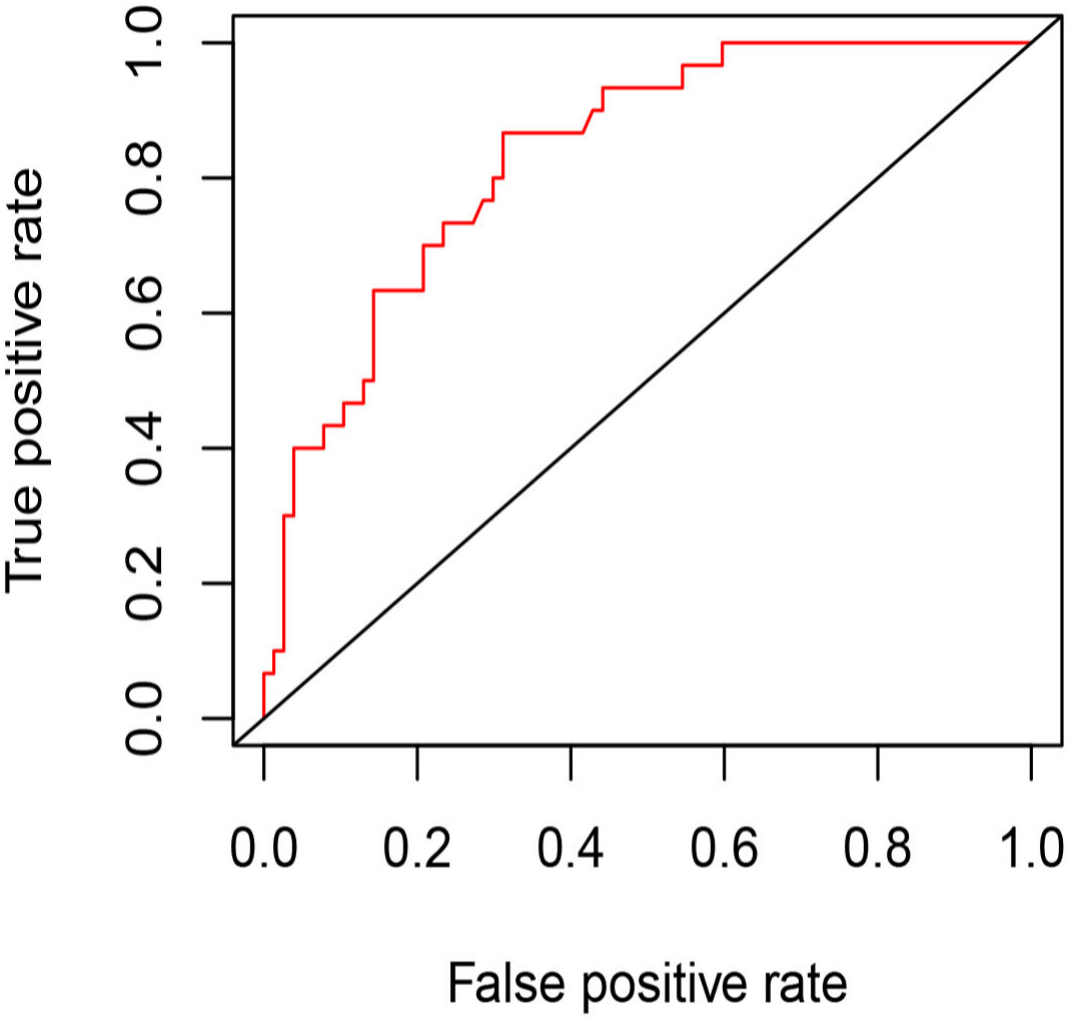
The ROC curve of the model for predicting residue of PCTNs after thermal ablation. AUC=0.832, optimal cutoff value=0.2 (specificity: 68.8%, sensitivity: 86.7%).

**Figure 7:**
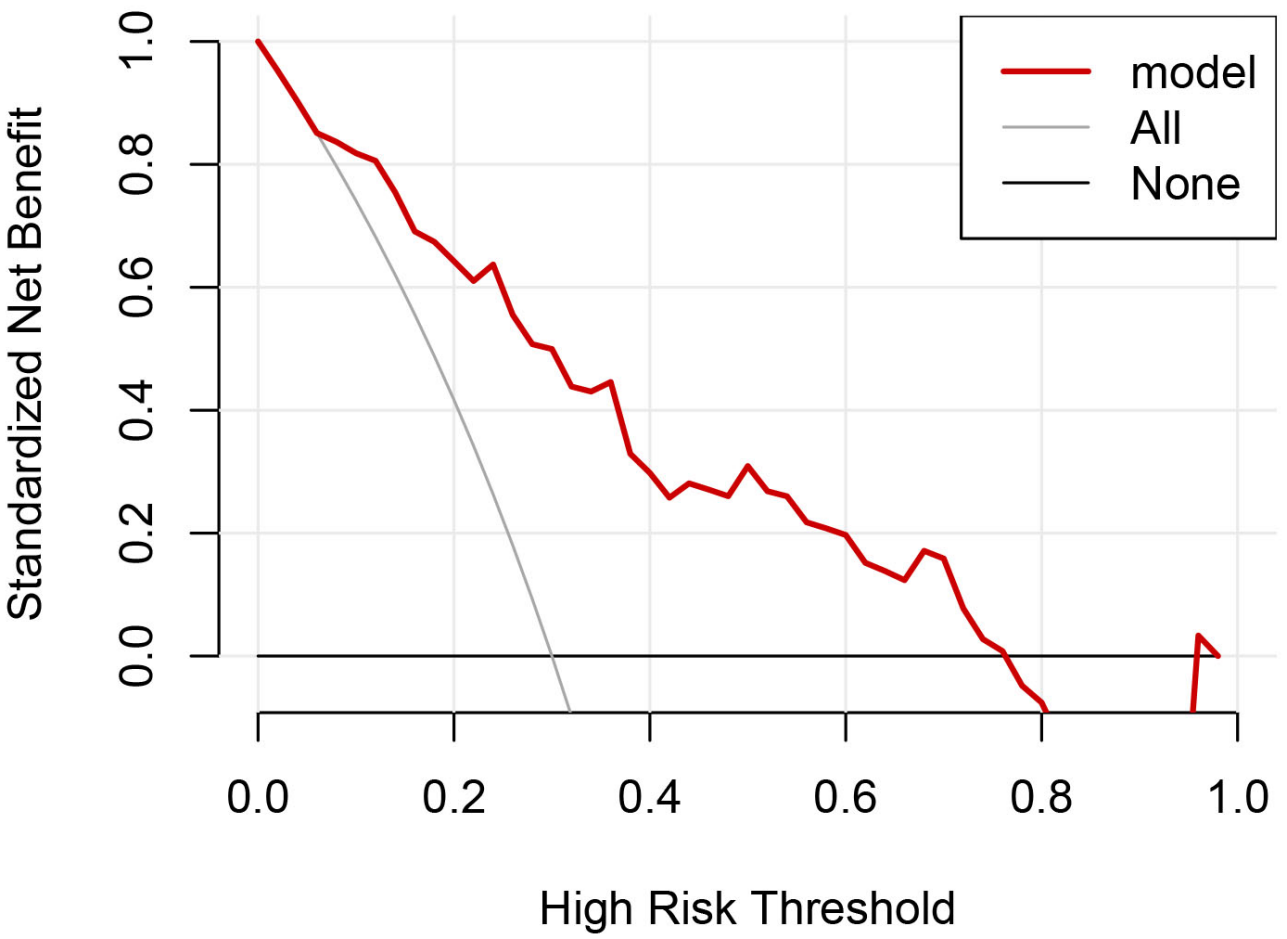
DCA of the model for predicting residue of PCTNs after thermal ablation.

Univariate and multivariate binary logistic regression analyses were conducted to identify factors associated with the residue of PCTNs after ablation. After univariate logistic regression analysis, the parameters with *P*<0.05 were selected into multivariate logistic regression analysis. As shown in **Table 3**, initial volume (*P*<0.001, OR=1.10) and septum (*P*=0.040, OR=2.97) were independent factors significantly associated with residue of PCTNs. Based on these two factors, a nomogram was established to predict the residue of PCTNs (**Figure 4**). Each factor had a corresponding score on the uppermost axis and the two scores were summed up to get a total point. Its location on the axis of the total point suggested a predicted residue rate. The p-value of the Hosmer–Lemeshow test was 0.639 >0.05 and indicated that the model fits well. A calibration curve was shown in **Figure 5**, which was closely fitted to the diagonal dotted line. It means the modal had a good ability of prediction. The C-index of the model was 0.832, equal to the AUC of the ROC curve (**Figure 6**). The optimal cutoff value by Youden method was 0.2 with a sensitivity of 86.7% and a specificity of 68.8%. Additionally, we performed internal stratified fivefold cross-validation to demonstrate the generalizability of the model (AUC =0.822, 0.750, 0.833, 0.900, 0.800; mean AUC=0.821). According to the decision curve analysis (DCA) of the model (**Figure 7**), we can see that at a risk threshold of 0.07-0.75, the net benefit of applying the nomogram was more than “treat all” and “treat none”, implying that the nomogram had a considerable clinical usefulness.

**Table 3:** Multivariate logistic regression analysis of predictors of residue of PCTNs after ablation.

Variables	OR (95%CI)	*P* Value
Initial volume(ml)	1.10 (1.05-1.16)	<0.001
Septum	2.97 (1.05-8.41)	0.040

## Discussion

In our study, we found that the initial volume and presence of septum inside the nodule were independent factors associated with residue of PCTNs after ethanol and thermal ablation. Then we developed a nomogram for predicting the probability of residue based on the two pre-ablative factors. The nomogram had good calibration, discrimination and clinical utility with an AUC of 0.832, indicating that it can identify patients with high risk of residue before therapy and improve treatment strategies. For the high-risk nodules, we should take aggressive ablation plan and intensive follow-up or even suggest for surgery.

Previous studies on efficacy of ablation of benign thyroid nodules focused on the volume reduction of treated nodule (23–25), probably because the main purpose of thermal ablation was to relieve compressive symptoms. Successful treatment was defined as a VRR≥50% after ablation. And regrowth was defined as an increase of the volume of the nodule≥50% of the minimum volume before ablation (12), which needs additional treatment. However, the nodule volume was influenced by aspiration of fluid and highly time-dependent. The rate of regrowth could increase with time until more than 5 years later (16, 18). This calls for long follow-up time to detect potential regrowth and cannot help time additional treatment early. In the study of Sim et al. (18), the total volume of nodule was composed of and affected by two parts according to US, the ablated volume (Va) and vital volume (Vv). During follow-up, Va would decrease gradually because of the absorption of necrotic tissue while Vv might increase due to regrowth. Therefore, the decrease of the nodule volume may consist of larger decrease of Va and smaller increase of Vv. Only tracing the change of total volume may miss an early sign of regrowth so the follow-up of Vv was more important in early detection of regrowth (3, 18, 26). The concept of Vv was consistent with the volume of residual vital tissue in our study. The widely used CEUS has a more reliable detection of residue (27) which was defined as enhanced area at ablative margin at one-month follow-up here. Zhao et al. found that nodules completely ablated had larger VRR compared to those incompletely ablated at 6-month follow-up, assessed with CEUS (28). Thus, in our study, we chose the presence of residual vital tissue as an outcome indicator.

In this study, we found that the larger the thyroid nodule was, the higher was the probability of residue after ablation. This finding was consistent with previous studies on predictive factors of efficacy of thyroid nodule ablation (15, 19, 29, 30). This was easy to understand because it was difficult to completely cauterize the margin of a large nodule in a single session. Meanwhile, the presence of septum was also associated with a higher residue rate. The presence of septum divided the nodule into several cysts containing fluid. Different from the cystic or solid nodules, the thermal ablation of PCTNs was conducted after simple aspiration of internal fluid with or without EA. Aspiration of fluid can not only greatly relieve the symptoms but was also helpful for a more effective ablation. For nodules with septum, it might be difficult to aspirate all the fluid completely. This fluid serves as a heat insulator, impeding the transmission of heat to all the parts of the nodule, especially those deeper than the septum. As a result, higher ablation power, longer duration and more release times of energy or multiple sessions were needed to achieve complete ablation. Thus the presence of septum was related to incomplete ablation or residue of the nodule regardless of the type of the septum. Moreover, we found that the adjacency to critical structures and vascularity were not associated to the residue of the PCTNs, which did not agree with a previous similar study (3). It was possibly because we adopted ethanol ablation as a combined therapy and hydrodissection to achieve a more complete ablation and avoid injury of the critical structures at the same time. Besides, our study focused on PCTNs so the heat-sink effect of vasculature was not so prominent and could be overcome by higher power.

There were several limitations in our study. First, it was a retrospective study. Second, this study was only conducted at single center with internal validation using stratified fivefold cross-validation. To assess the generalizability of the model more reliably, external validation was needed. Third, the follow-up time of some patients was short so we could not obtain the long-term VRR which was more convincing than short-term VRR to validate the correlation between short-term residue and long-term regrowth of nodules.

## Conclusion

In this study, we developed a nomogram model for the pre-ablative prediction of residue of PCTNs undergoing ethanol and thermal ablation based on initial volume and presence of septum in the nodules. The model could discriminate patients with high or low residue possibility with good calibration. It was useful in the decision of treatment and follow-up for different patients in clinical practice.

## Data availability statement

The raw data supporting the conclusions of this article will be made available by the authors, without undue reservation.

## Ethics statement

The studies involving human participants were reviewed and approved by IEC for Clinical Research and Animal Trials of the First Affiliated Hospital of Sun Yat-sen University. Written informed consent for participation was not required for this study in accordance with the national legislation and the institutional requirements. Written informed consent was not obtained from the individual(s) for the publication of any potentially identifiable images or data included in this article.

## Author contributions

DL and XZ interpreted the data and wrote the manuscript. YZ produced figures and tables. TH, RZ and WZ collected the patient data and performed the statistical analysis. XX and MX designed the study and performed ablation procedure. DL and XZ contributed equally to this work and share first authorship. All authors contributed to the article and approved the submitted version.
